# Heterogeneous Determinants of Quality of Life in Different Phenotypes of Parkinson’s Disease

**DOI:** 10.1371/journal.pone.0137081

**Published:** 2015-09-03

**Authors:** Seyed-Mohammad Fereshtehnejad, Mahdiyeh Shafieesabet, Farzaneh Farhadi, Hasti Hadizadeh, Arash Rahmani, Nader Naderi, Dena Khaefpanah, Gholam Ali Shahidi, Ahmad Delbari, Johan Lökk

**Affiliations:** 1 Division of Clinical geriatrics, Department of Neurobiology, Care Sciences, and Society (NVS), Karolinska Institutet, Stockholm, Sweden; 2 Firoozgar Clinical Research Development Center (FCRDC), Firoozgar Hospital, Iran University of Medical Sciences, Tehran, Iran; 3 Medical Student Research Committee (MSRC), Faculty of Medicine, Iran University of Medical Sciences, Tehran, Iran; 4 Mental Health Research Center, Tehran Institute of Psychiatry, School of Behavioral Sciences and Mental Health, Iran University of Medical Sciences, Tehran, Iran; 5 Movement disorders clinic, Department of neurology, Faculty of Medicine, Iran University of Medical Sciences, Tehran, Iran; 6 Iranian Research Center on Aging, University of Social Welfare and Rehabilitation, Tehran, Iran; 7 Department of Geriatric Medicine, Karolinska University Hospital, Stockholm, Sweden; Hospital General Dr. Manuel Gea González, MEXICO

## Abstract

**Objectives:**

Health-related quality of life (HRQoL) is considered a very important outcome indicator in patients with Parkinson’s disease (PD). A broad list of motor and non-motor features have been shown to affect HRQoL in PD, however, there is a dearth of information about the complexity of interrelationships between determinants of HRQoL in different PD phenotypes. We aimed to find independent determinates and the best structural model for HRQoL, also to investigate the heterogeneity in HRQoL between PD patients with different phenotypes regarding onset-age, progression rate and dominant symptom.

**Methods:**

A broad spectrum of demographic, motor and non-motor characteristics were collected in 157 idiopathic PD patients, namely comorbidity profile, nutritional status, UPDRS (total items), psychiatric symptoms (depression, anxiety), fatigue and psychosocial functioning through physical examination, validated questionnaires and scales. Structural equation model (SEM) and multivariate regressions were applied to find determinants of Parkinson’s disease summary index (PDSI) and different domains of HRQoL (PDQ-39).

**Results:**

Female sex, anxiety, depression and UPDRS-part II scores were the significant independent determinants of PDSI. A structural model consisting of global motor, global non-motor and co-morbidity indicator as three main components was able to predict 89% of the variance in HRQoL. In older-onset and slow-progression phenotypes, the motor domain showed smaller contribution on HRQoL and the majority of its effects were mediated through non-motor features. Comorbidity component was a significant determinant of HRQoL only among older-onset and non-tremor-dominant PD patients. Fatigue was not a significant indicator of non-motor component to affect HRQoL in rapid-progression PD.

**Conclusions:**

Our findings showed outstanding heterogeneities in the pattern and determinants of HRQoL among PD phenotypes. These factors should be considered during the assessments and developing personalized interventions to improve HRQOL in PD patients with different phenotypes or prominent feature.

## Introduction

Both the chronic nature and the wide spectrum of Parkinson’s disease (PD) manifestations affect several aspects of patients’ daily life. Thus, health-related quality of life (HRQoL) has been considered as an important outcome indicator for management, care and progression of PD[1]. Quite recently, many studies have investigated the impact of different variables on HRQoL in PD patients including disease severity, motor symptoms, non-motor symptoms, nutritional status, demographic and socioeconomic characteristics[1,2,3,4,5,6,7,8,9]. Yet, a few of them have included the broad range of parkinsonian features all together and compare their independent role and strength of their affect on HRQoL. On the other hand, patients with PD show significant heterogeneity in their motor and non-motor features[10], which is a great obstacle in generalizability of the pattern of HRQoL for PD patients with different phenotypes. While the National Institutes of Health has recently delineated subtype-identification as one of the top priorities in the field of PD clinical research[11], there is a dearth of information about determinants of QoL in different PD subtypes.

Having evaluated a broad spectrum of baseline, sociodemographic, nutritional, motor and non-motor features of PD, we intended to apply advanced statistical methods namely multivariate regressions and structural equation modeling (SEM) to: 1) investigate the factors that affect HRQoL in PD patients, 2) compare their independence and strength of their effects on HRQoL, 3) model HRQoL with the best hypothesized structural model and 4) explore the structural heterogeneity in the optimum model for HRQoL within different PD phenotypes.

## Materials and Methods

### Study Setting & Subjects

This study was conducted on 157 patients with idiopathic Parkinson’s disease (IPD), which were consecutively recruited from an outpatient referral movement disorder clinic in Tehran, Iran. The ethics committee of the neurology department at Firoozgar Clinical Research Development Center (FCRDC) approved the study protocol and informed consent was verbally obtained from all participants. Since this project was designed as an observational study and no intervention was applied for research purposes, the verbal form of consent was approved by the aforementioned ethics committee according to their instructions. Following inclusion criteria were applied to enroll the patients: diagnosis of IPD based on the UK brain bank criteria[12], current age ≥35 years, and to be cognitively eligible to validly answer the questionnaires. Cognitive status was judged through interview by the neurologist and patients with severe dementia who gave invalid answers were excluded.

Three phenotyping approaches were used to divide study participants into different subgroups as follows:

- Onset-age: younger-onset (n = 50, diagnostic age ≤50 *yr*) versus older-onset (n = 106, diagnostic age >50 *yr*)
- Progression: slow (n = 95) versus rapid (n = 40) based on the clustering solution recommended by *Gasparoli et al* using Unified Parkinson's Disease Rating Scale (UPDRS)-Part II, UPDRS-Part III, dyskinesia and motor fluctuations as the clustering characteristics [13]
- Dominant symptom: tremor (n = 76) versus non-tremor (n = 75) based on the median value of the tremor motor score (cut-off value = 13.3)

### Assessments

Data collection was performed through interviews with eligible patients by a trained group of medical interns to fill validated questionnaires and scales. One neurologist specialized in movement disorders examined all patients for clinical assessment and diagnosis. Demographic and baseline data were collected by means of a checklist. Assessment of clinical characteristics and HRQoL were performed during the “On” status consisting of:

### Motor Severity

a Unified Parkinson's Disease Rating Scale (UPDRS) subscales I—IV
b Hoehn and Yahr (H & Y) staging
c Schwab and England activities of daily living (ADL)
d Dyskinesia score: sum of UPDRS-Part IV items 32–34
e Fluctuation score: sum of UPDRS-Part IV items 36–39

### Motor Subtypes

a Postural-instability-gait-difficulty (PIGD) score: sum of UPDRS-Part III items concerning rise, gait, and postural instability
b FOSS score: sum of UPDRS-Part II items on freezing, speech and swallowing
c Predominance of core manifestations: proportion of UPDRS-Part III ‘‘on” motor scores accounted for tremor (items 20–21), rigidity (item 22), bradykinesia (items 23–26 and 31), and gait (items 27–30) in percentage
d Asymmetry index: absolute differences in UPDRS between sides divided by the total UPDRS III (0 = perfect symmetry, 1 = absolute asymmetry)
e Axial/limb ratio: sum of UPDRS-Part III items 18, 19, 22 and 27–30 divided by sum of UPDRS-Part III items 20–26
f Presence of falls and freezing

### Non-Motor Manifestations

a Depression: using the Persian-translated and validated version of the Hospital Anxiety and Depression Score (HADS)[14] consisting of 7 items for depression scored between 0 to 21 where higher scores show more severe depressive symptoms
b Anxiety: assessed by HADS similar to depression
c Hallucinations/Illusions: evaluated using UPDRS-Part I, item 2
d Apathy: evaluated using UPDRS-Part I, item 4
e Fatigue: by means of the Persian-translated and validated version of the Fatigue Severity Scale (FSS) [15], which includes 9 questions with a total score ranging from 0 to 7 (higher scores correspond more severe fatigue)
f Nutritional status: using the Persian-translated and validated version of the Mini Nutritional Assessment (MNA)[16] including anthropometric measurements of body mass index (BMI), body mass index (BMI), arm and calf circumferences totally scoring between 0 to 30 where higher scores demonstrate better status
g Psychosocial functioning: evaluated by the Persian-translated and validated version of the scales for outcomes in Parkinson's disease-psychosocial questionnaire (SCOPA-PS)[17], which consists of 11 items and a summary index that is scored between 0 to 100% (higher score corresponds a worse condition)

### Health-Related Quality of Life (HRQoL)

Validated Persian version of the 39-item PD questionnaire (PDQ-39)[18] was applied to assess HRQoL. Other than domain-specific scores, PD summary index (PDSI) was calculated as the mean score of all domains. PDSI varies between 0 to 100% and a higher score indicates poorer HRQoL in PD patients.

Detailed participant-level information on all variables and measurements is presented in S1 Text.

### Statistical Analysis

For description of numerical variables, mean and standard deviation (SD) were used except for discrete values where median and interquartile range (IQR) was reported. We applied univariate linear regression to calculate beta correlation coefficients and their 95% confidence interval (CI) between baseline and clinical characteristics with the numeric PDSI score. Prior to perform further analyses and in order to avoid case-wise deletion and decreased statistical power, 95 single missing values (0.25% of the whole datasheet) were imputed using multiple regressions. Two-step cluster analysis was used to implement the clustering solution recommended by *Gasparoli et al*[13] to divide PD patients into slow-progression versus rapid-progression phenotypes. Multivariate linear regression was performed using the list of significant univariate demographic variables for adjustment, and the best representative variables from motor severity, motor subtypes and non-motor assessments. We avoided including correlated indicators of one single entity. Tolerance index representing the proportion of variance for each independent variable that are not explained by other independent variables in the model, were calculated and were considered acceptable if >0.4. Conventionally, the variance inflation factor (VIF) (1/tolerance) was also reported and aimed to be <2.5 to avoid collinearity in the models. If the collinearity occurred, then the variable with higher tolerance and larger standardized coefficient was kept and the other collinear variable was deleted from the regression model.

Furthermore, a structural equation model (SEM) was applied to construct a hypothetical model with three latent variables representing global motor, non-motor and HRQoL components. After structural modifications, standardized regression weight (SRW) for each included component was reported. Fitness of each SEM was assessed using the absolute fit indices consisting of Normed Fit Index (NFI), Comparative Fit Index (CFI), Tucker-Lewis Index (TLI) and Root Mean Square Error of Approximation (RMSEA). An NFI, CFI and TLI value between 0.06 and 0.08 and RMSEA <0.08 indicate an acceptable model fit[19].

## Results

### Baseline Characteristics

Study participants consisted of 157 IPD patients, 108 (68.8%) males and 49 (31.2%) females, with the mean age of 61.4 (SD = 11.2) *yr* at the time of enrollment and average PD duration of 6.8 (SD = 5.2) *yr*. The mean of total UPDRS score was 32.2 (SD = 18.1) and the median Hoehn and Yahr stage was 2 (IQR = 1.5). Other demographic and clinical characteristics including motor severity, motor subtype, non-motor assessments and HRQoL scores are listed in Table 1. The worst dimension-specific scores of the PDQ-39 questionnaire were observed in the “*emotional well-being*” [28.4 (SD = 23.6)] and “*mobility*” [28.2 (SD = 26.4)] domains and the average value of the PDSI was 21.2 (SD = 15.4).

**Table 1:** Baseline and clinical characteristics of the Parkinson’s disease patients (n = 157).

Characteristics	Value
**Demography and General Information**	
**Age**-year *(mean ± SD)*	
*Current*	61.4 ± 11.2
*At disease onset*	54.7 ± 11.9
**Gender** *NO (%)*	
Female	49 (31.2)
Male	108 (68.8)
**Level of Education** *NO (%)*	
Illiterate	17 (11.0)
Primary and/ or secondary	37 (23.9)
High school/diploma	43 (27.7)
College and/ or university	58 (37.4)
**Comorbidities** *NO (%)*	
Hypertension	28 (18.1)
Ischemic heart disease	24 (15.7)
Osteoarthritis	19 (12.4)
Diabetes	20 (13.1)
Stroke/Transient ischemic attack	1 (0.7)
Chronic obstructive pulmonary disease	1 (0.7)
Total score *(mean ± SD)*	0.6 ± 1.0
**Duration of Parkinson’s Disease**-year *(mean ± SD)*	6.8 ± 5.2
**Levodopa Dose**-mg *(mean ± SD)*	
Cumulative daily dose	864.7 ± 447.8
Weight-adjusted daily dose	12.6 ± 7.2
**Motor Severity**	
**UPDRS Score** *(mean ± SD)*	
*Part II*- ADL	11.7 ± 7.4
*Part III*- Motor	15.5 ± 9.2
*Part IV*- Complications	3.4 ± 2.8
Dyskinesia	0.9 ± 1.7
Fluctuations	1.7 ± 1.3
Total score	32.2 ± 18.1
**Hoehn and Yahr Stage** *median (IQR)*	2 (1.5)
**Schwab and England Activities of Daily Living Score**-(%) *(mean ± SD)*	80.5 ± 18.0
**Motor Impairment Score A^1^** *(mean ± SD)*	11.5 ± 6.3
**Motor Impairment Score B^2^** *(mean ± SD)*	4.0 ± 3.6
**Motor Subtypes**	
**Tremor**-% of UPDRS-Part III *(mean ± SD)*	14.9 ± 13.7
**Rigidity**-% of UPDRS-Part III *(mean ± SD)*	10.2 ± 7.2
**Bradykinesia**-% of UPDRS-Part III *(mean ± SD)*	41.3 ± 17.3
**Gait**-% of UPDRS-Part III *(mean ± SD)*	17.7 ± 12.2
**Freezing** *N (%)*	54 (34.4)
**Falls** *N (%)*	62 (39.5)
**Axial/Limb Ratio** *(mean ± SD)*	0.9 ± 0.7
**Asymmetry Index** *(mean ± SD)*	0.2 ±0.2
**PIGD Score** *(mean ± SD)*	2.3 ± 2.4
**FOSS Score** *(mean ± SD)*	1.7 ± 1.7
**Non-Motor Assessments**	
**UPDRS Score** *(mean ± SD)*	
*Part I*- Mental	2.1 ± 2.4
**Cognitive Impairment** *N (%)*	47 (29.9)
**Hallucination** *N (%)*	19 (12.1)
**Apathy** *N (%)*	58 (37.2)
**Sleep Disturbances** *N (%)*	58 (36.9)
**Orthostasis** *N (%)*	28 (17.8)
**Anxiety Score (HADS)** *(mean ± SD)*	6.8 ± 5.1
**Depression Score (HADS)** *(mean ± SD)*	5.1 ± 4.4
**Fatigue Score (FSS)** *(mean ± SD)*	4.5 ± 1.9
**Nutritional Status (MNA)** *(mean ± SD)*	
Screening score	12.7 ± 2.0
Assessment score	12.6 ± 1.9
Total score	25.2 ± 3.3
**Psychosocial Functioning Score (SCOPA-PS)**-(%) *(mean ± SD)*	25.9 ± 22.3
**Health-Related Quality of Life (HRQoL)**	
**Quality of Life (PDQ-39)** *(mean ± SD)*	
*Dimension I*-Mobility	28.2 ± 26.4
*Dimension II*-Activity of daily living (ADL)	26.4 ± 25.6
*Dimension III*-Emotional well-being	28.4 ± 23.6
*Dimension IV*-Stigma	21.9 ± 25.2
*Dimension V*-Social support	11.5 ± 20.3
*Dimension VI*-Cognitions	17.9 ± 20.1
*Dimension VII*-Communication	15.1 ± 19.5
*Dimension VIII*-Bodily Discomfort	22.1 ± 23.0
**Parkinson’s disease summary index (PDSI)** *(mean ± SD)*	21.2 ± 15.4

SD: standard deviation; IQR: interquartile range; PIGD: postural-instability-gait-difficulty; FOSS: freezing-speech-swallowing

^1^ Score A is the sum of UPDRS-Part III items concerning facial expression, tremor, rigidity, and Bradykinesia which are considered relatively L-dopa responsive

^2^ Score B is the sum of UPDRS-Part III items concerning speech and axial impairment (arising from chair, posture, postural stability, gait) which are considered relatively L-dopa non-responsive.

### Univariate Associations

Table 2 shows the univariate linear correlations between baseline characteristics, motor severity and subtypes, and non-motor scales with PDSI as a single indicator of HRQoL in the whole study participants. Among demographic characteristics, female sex [unadjusted coefficient = 10.40 (95%CI: 5.41–15.38)], lower education [unadjusted coefficient = -5.03 (95%CI: -7.26 – -2.80)], higher number of comorbidities [unadjusted coefficient = 3.07 (95%CI: 0.59–5.54)] and higher weight-adjusted daily levodopa dose [unadjusted coefficient = 0.48 (95%CI: 0.16–0.80)] were all related to higher PDSI. Several motor and non-motor variables including all parts of UPDRS, psychiatric symptoms, fatigue and psychosocial functioning were significantly correlated with the PDSI (Table 2).

**Table 2:** Univariate linear correlations between the baseline and clinical characteristics with Parkinson’s disease summary index (PDSI) of the health-related quality of life (HRQoL) (n = 157).

Characteristics	Univariate Coefficient β *(95% confidence interval)*	*p*-value
**Demography**		
**Onset Age**	-0.05 (-0.25–0.16)	0.655
**Female Sex**	10.40 (5.41–15.38)	<0.001*
**Level of Education**	-5.03 (-7.26 – -2.80)	<0.001*
**Comorbidity Score^1^**	3.07 (0.59–5.54)	0.016*
**Duration of Parkinson’s Disease**	0.56 (0.10–1.02)	0.017*
**Levodopa Treatment**		
Cumulative daily dose	0.01 (0–0.01)	0.064
Weight-adjusted daily dose	0.48 (0.16–0.80)	0.004*
**Motor Severity**		
**UPDRS Score**		
*Part II*- ADL	1.34 (1.08–1.59)	<0.001*
*Part III*- Motor	0.71 (0.47–0.95)	<0.001*
*Part IV*		
Dyskinesia score	1.18 (-0.23–2.59)	0.100
Fluctuations score	0.09 (-1.83–2.01)	0.926
Total score	0.53 (0.41–0.64)	<0.001*
**Hoehn and Yahr Stage**	6.16 (3.61–8.70)	<0.001*
**Schwab and England Activities of Daily Living Score**	-0.48 (-0.59 – -0.37)	<0.001*
**Motor Impairment Score A^2^**	0.93 (0.57–1.29)	<0.001*
**Motor Impairment Score B^3^**	1.71 (1.07–2.34)	<0.001*
**Motor Subtypes**		
**Tremor**-% of UPDRS-Part III	-0.26 (-0.44 – -0.08)	0.006*
**Rigidity**-% of UPDRS-Part III	-0.42 (-0.77 – -0.08)	0.017*
**Bradykinesia**-% of UPDRS-Part III	0.12 (-0.03–0.26)	0.108
**Gait**-% of UPDRS-Part III	0.19 (-0.02–0.39)	0.077
**Freezing**	13.33 (8.67–18.00)	<0.001*
**Falls**	12.15 (7.56–16.74)	<0.001*
**Axial/Limb Ratio**	0.03 (-3.37–3.43)	0.984
**Asymmetry Index**	-18.91 (-29.10 – -8.72)	<0.001*
**PIGD Score**	2.34 (1.38–3.31)	<0.001*
**FOSS Score**	4.65 (3.43–5.88)	<0.001*
**Non-Motor Assessments**		
**UPDRS Score**		
*Part I*- Mental	3.63 (2.74–4.51)	<0.001*
**Cognitive Impairment**	7.98 (2.82–13.14)	0.003*
**Hallucination**	16.66 (9.68–23.63)	<0.001*
**Apathy**	12.75 (8.12–17.39)	<0.001*
**Sleep Disturbances**	8.93 (4.09–13.77)	<0.001*
**Orthostasis**	3.52 (-2.82–9.85)	0.274
**Anxiety Score (HADS)**	1.75 (1.36–2.14)	<0.001*
**Depression Score (HADS)**	2.47 (2.07–2.87)	<0.001*
**Fatigue Score (FSS)**	3.63 (2.47–4.79)	<0.001*
**Nutritional Status (MNA)**		
Screening score	-2.37 (-3.45 – -1.29)	<0.001*
Assessment score	-4.39 (-5.40 – -3.39)	<0.001*
Total score	-2.41 (-3.05 – -1.77)	<0.001*
**Psychosocial Functioning Score (SCOPA-PS)**	0.55 (0.48–0.62)	<0.001*

PIGD: postural-instability-gait-difficulty; FOSS: freezing-speech-swallowing

* Statistical significant correlation (two-tailed *p*-value<0.05)

^1^ Total number of comorbidities

^2^ Score A is the sum of UPDRS-Part III items concerning facial expression, tremor, rigidity, and Bradykinesia which are considered relatively L-dopa responsive

^3^ Score B is the sum of UPDRS-Part III items concerning speech and axial impairment (arising from chair, posture, postural stability, gait) which are considered relatively L-dopa non-responsive.

### Multivariate Regression

The results from multivariate linear regression models to find the independent predictors of HRQoL evaluated by the PDSI and within different dimensions of the PDQ-39 are summarized in Table 3. Using sex, level of education, comorbidity score and PD duration as the baseline covariates, anxiety [adjusted coefficient = 0.51 (95%CI: 0.15–0.87)], depression [adjusted coefficient = 1.11 (95%CI: 0.63–1.59)] and UPDRS-part II (ADL) [adjusted coefficient = 0.90 (95%CI: 0.60–1.20)] scores were the significant independent determinants of PDSI. In dimension-specific analysis, female sex, higher depression score and more impaired ADL (UPDRS-Part II) were shown to be the statistically significant predictors of worse HRQoL in “*mobility*” and “*social support*” domains (all adjusted coefficients >0 and *p*<0.05). “*Emotional well-being*” was significantly worse in IPD patients who were female [adjusted coefficient = 7.18 (95%CI: 1.02–13.34)], scored higher for anxiety [adjusted coefficient = 1.65 (95%CI: 0.98–2.31)] and depression [adjusted coefficient = 1.99 (95%CI: 1.11–2.88)] and had less severe motor signs (UPDRS-Part III) [adjusted coefficient = -0.53 (95%CI: -0.93 – -0.12)]. Furthermore, higher number of comorbidities [adjusted coefficient = 2.63 (95%CI: 0.02–5.24)] accompanied with a worse “*cognition*” dimension of the HRQoL in IPD patients.

**Table 3:** Multivariate linear regression models to find the baseline and clinical predictors of the Parkinson’s disease summary index (PDSI) and different dimensions of the health-related quality of life (HRQoL) in Parkinson’s disease patients (n = 157).

Independent Variables	Unstandardized Coefficients	Standardized Coefficients	*p*-value	Collinearity Statistics	
				*Tolerance*	*VIF*
**Parkinson’s disease summary index (PDSI)** *(whole model)*					
**Female Sex**	5.30 (1.97–8.62)	0.16	0.002*	0.83	1.21
**Disease Duration**	-0.24 (-0.54–0.07)	-0.08	0.127	0.78	1.29
**Comorbidity Score**	1.33 (-0.15–2.81)	0.08	.079	0.97	1.03
**Anxiety Score**	0.51 (0.15–0.87)	0.17	.006*	0.59	1.70
**Depression Score**	1.11 (0.63–1.59)	0.32	<0.001*	0.45	2.21
**MNA Total Score**	-0.14 (-0.71–0.43)	-0.03	0.634	0.57	1.77
**Fatigue Score**	0.33 (-0.58–1.24)	0.04	0.48	0.67	1.50
**UPDRS Score**					
*Part I-items 1*, *2*, *4*	0.69 (-0.20–1.57)	0.08	0.127	0.71	1.41
*Part II*- ADL	0.90 (0.60–1.20)	0.43	<0.001*	0.41	2.42
*Part III*- Motor	-0.12 (-0.34–0.10)	-0.07	0.269	0.49	2.05
**Constant**	3.66 (-13.40–20.72)	-	.672	-	-
***Dimension I*-Mobility** *(significant predictors)*					
**Female Sex**	10.86 (4.12–17.60)	0.19	0.002*	0.83	1.21
**Depression Score**	1.41 (0.44–2.38)	0.23	0.005*	0.45	2.21
**UPDRS Score**					
*Part II*- ADL	1.19 (0.59–1.80)	0.33	<0.001*	0.41	2.42
***Dimension II*-Activity of daily living (ADL)** *(significant predictors)*					
**Depression Score**	1.26 (0.31–2.21)	0.22	0.009*	0.45	2.21
**UPDRS Score**					
*Part II*- ADL	1.98 (1.39–2.57)	0.57	<0.001*	0.41	2.42
***Dimension III*-Emotional well-being** *(significant predictors)*					
**Female Sex**	7.18 (1.02–13.34)	0.14	0.023	0.83	1.21
**Anxiety Score**	1.65 (0.98–2.31)	0.36	<0.001*	0.59	1.70
**Depression Score**	1.99 (1.11–2.88)	0.37	<0.001*	0.45	2.21
**UPDRS Score**					
*Part III*- Motor	-0.53 (-0.93 – -0.12)	-0.20	<0.012*	0.49	2.05
***Dimension IV*-Stigma** *(significant predictors)*					
**Anxiety Score**	1.15 (0.20–2.10)	0.23	0.018*	0.59	1.70
**UPDRS Score**					
*Part II*- ADL	0.85 (0.07–1.64)	0.25	0.034*	0.41	2.42
*Part III*- Motor	-0.57 (-1.15–0.01)	-0.21	0.055*	0.49	2.05
***Dimension V*-Social support** *(significant predictors)*					
**Female Sex**	10.44 (3.77–17.12)	0.24	0.002*	0.83	1.21
**Depression Score**	1.46 (0.50–2.42)	0.31	0.003*	0.45	2.21
**UPDRS Score**					
*Part II*- ADL	0.72 (0.12–1.31)	0.26	0.019*	0.41	2.42
***Dimension VI*-Cognitions** *(significant predictors)*					
**Comorbidity Score**	2.63 (0.02–5.24)	0.13	0.048*	0.97	1.03
**Anxiety Score**	0.62 (-0.01–1.25)	0.16	0.055*	0.59	1.70
**UPDRS Score**					
*Part I-items 1*, *2*, *4*	3.85 (2.29–5.40)	0.36	<0.001*	0.71	1.41
*Part II*- ADL	1.03 (0.50–1.55)	0.38	<0.001*	0.41	2.42
***Dimension VII*-Communication** *(significant predictors)*					
**Depression Score**	1.33 (0.49–2.17)	0.30	0.002*	0.45	2.21
**UPDRS Score**					
*Part II*- ADL	0.81 (0.29–1.33)	0.31	0.002*	0.41	2.42
***Dimension VIII*-Bodily Discomfort** *(significant predictors)*					
**Female Sex**	11.12 (3.97–18.27)	0.23	0.003*	0.83	1.21
**UPDRS Score**					
*Part I-items 1*, *2*, *4*	2.28 (0.38–4.18)	0.19	0.019*	0.71	1.41

All models were performed using the independent/covariate variables listed in the first model for Parkinson’s disease summary index (PDSI) as dependent outcome.

### Structural Equation Modeling

**Fig 1:**
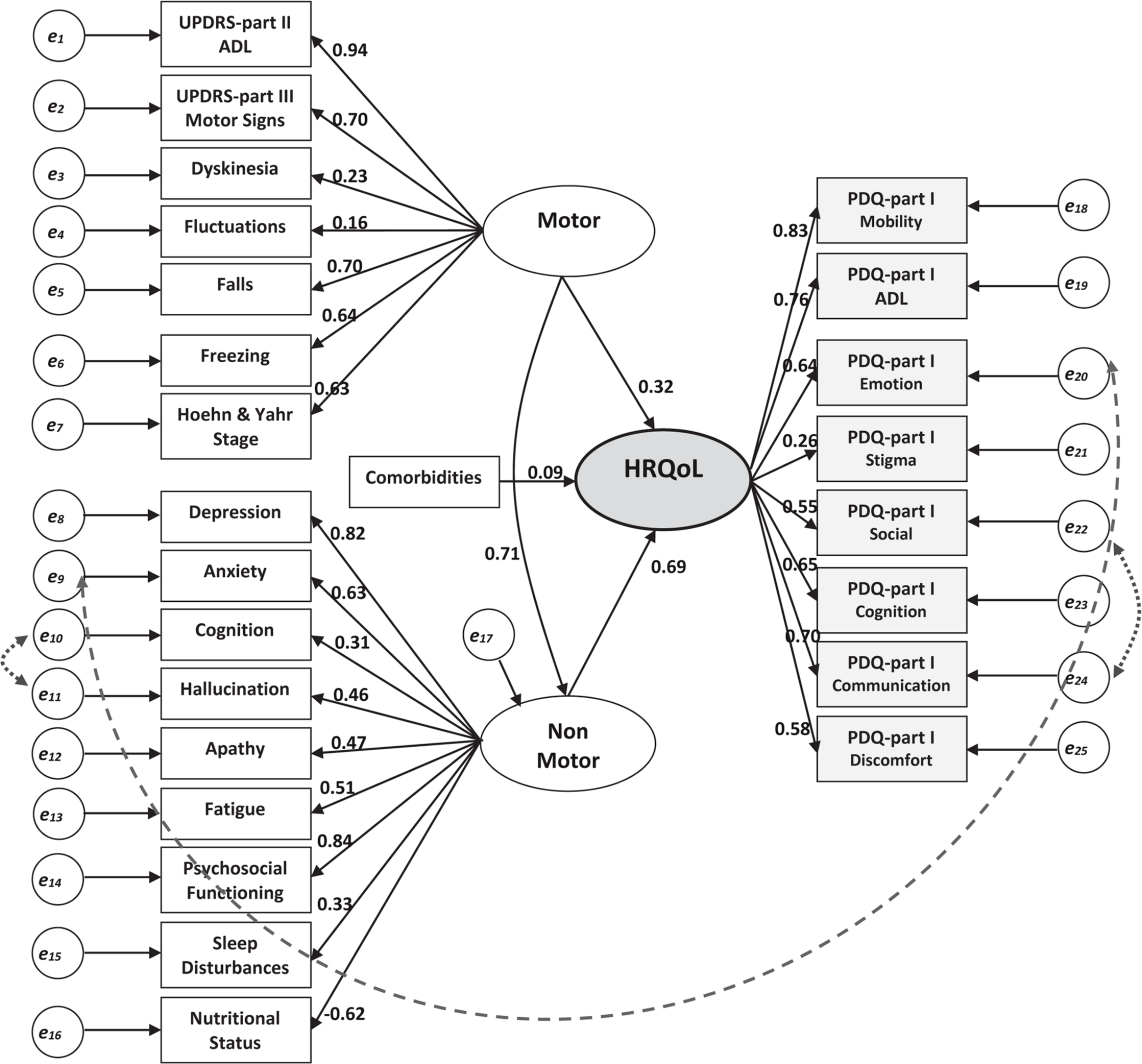
Structural equation model for the factors affecting health-related quality of life (HRQoL) in patients with Parkinson’s disease [all standardized regression weights are statistically significant at p<0.001 except for the effect of comorbidities on HRQoL (p = 0.045), the indication of motor domain on fluctuations (p = 0.062) and dyskinesia (p = 0.001), and the indication of HRQoL on stigma (p = 0.001)]

Fig 1 illustrates the hypothetical structural model for the factors affecting HRQoL in IPD patients. With RMSEA = 0.08, NFI = 0.74, CFI = 0.84 and TLI = 0.82, the model could acceptably explain 89% of the variance of HRQoL. Three direct correlations (cognition and hallucination in the non-motor section, anxiety on “*emotional well-being*” dimension of HRQoL, “*social support*” and “*communication*” dimensions of HRQoL) were added in order to improve the model. Among the entire study samples, non-motor latent domain had a larger direct effect on HRQoL compared to that of the motor (SRW = 0.69 vs. 0.32), while the motor domain showed an indirect effect mediated through the non-motor section as large as 0.49 resulting in the total effect of 0.81. UPDRS-Part II (ADL) (SRW = 0.94), UPDRS-Part III (motor signs) (SRW = 0.70) and falling (SRW = 0.70) were the strongest indicators of the motor latent variable. Psychosocial functioning (SRW = 0.84), depression (SRW = 0.82) and anxiety (SRW = 0.63) were the most determinant factors for the non-motor latent domain to affect HRQoL.

The same hypothetical SEM was rerun in subgroups of PD with different phenotypes. As it is shown in Table 4, motor domain showed larger direct impact on HRQoL compared to non-motor section in younger-onset (SRW = 0.61, *p*<0.001)and rapid-progression PD (SRW = 0.57, *p*<0.001), whereas in other phenotypes non-motor domain had larger direct effect (all *p*<0.05). Among patients with slow-progression PD, non-motor domain showed the largest direct effect (SRW = 0.95, *p* = 0.005) on HRQoL and all of the effects of motor domain was mediated through non-motor section. Even though small, comorbid conditions showed significant effect on HRQoL among those with >50 years of age at the time of diagnosis (SRW = 0.14, *p* = 0.007) and non-tremor-dominant PD (SRW = 0.13, *p* = 0.037). In younger-onset patients, sleep disorder was not an important indicator of the non-motor latent variable, while depression was its strongest driver (SRW = 0.91, *p* = 0.004).Opposite to slow-progression phenotype, fatigue was shown to be a non-significant indicator for non-motor domain among those categorized as rapid-progression. In contrast to all other phenotypes, both dyskinesia (SRW = 0.42, *p* = 0.012) and fluctuations (SRW = 0.33, *p* = 0.043) remained significant indicators for motor domain in the younger-onset patients. “Stigma” was found to be an important indicator for HRQoL only in older-onset (SRW = 0.39, *p*<0.001), slow-progression (SRW = 0.33, *p* = 0.003) and non-tremor-dominant (SRW = 0.49, *p*<0.001) subgroups. “Cognition” had the highest SRW as an indicator of HRQoL among the older-onset patients (0.72), whereas “*mobility*” showed the largest SRW in younger-onset phenotype (0.90).

**Table 4:** Standardized direct, indirect and total regression weights from the structural equation models (SEM) to evaluate factors affecting health-related quality of life (HRQoL) in different phenotypes of Parkinson’s disease patients [Values are presented as standardized regression weight (*p*-value).]

Main Associations	**Exogenous variable**	**➔**	**Endogenous variable**	**Onset-Age**		**Progression**		**Dominant Symptom**	
	**(Independent)**		**(Dependent)**						
				***≤50 yr***	***>50 yr***	***Slow***	***Rapid***	***Tremor***	***Non-Tremor***
				***(n = 50)***	***(n = 106)***	***(n = 95)***	***(n = 40)***	***(n = 76)***	***(n = 75)***
	Motor Domain	*Direct*	HRQoL	0.61 (<0.001)	0.21 (0.049)	-	0.57 (<0.001)	0.29 (0.018)	0.23 (0.053)
		*Indirect*	HRQoL	0.25	0.60	0.60	0.29	0.50	0.57
		*Total*	HRQoL	0.86	0.81	0.60	0.86	0.79	0.80
	Non-Motor Domain	➔	HRQoL	0.43 (0.019)	0.78 (<0.001)	0.95 (0.005)	0.53 (0.031)	0.68 (<0.001)	0.79 (<0.001)
	Comorbidities	➔	HRQoL	-	0.14 (0.007)	-	-	-	0.13 (0.037)
	Motor Domain	➔	Non-Motor Domain	0.59 (0.032)	0.77 (<0.001)	0.63 (0.012)	0.54 (0.049)	0.73 (<0.001)	0.73 (<0.001)
Indicators of latent variables	Motor Domain	➔	UPDRS-ADL	0.92 (<0.001)	0.93 (<0.001)	0.85 (<0.001)	0.92 (<0.001)	0.90 (<0.001)	0.98 (<0.001)
		➔	UPDRS-Motor Signs	0.80 (<0.001)	0.67 (<0.001)	0.41 (<0.001)	0.62 (<0.001)	0.78 (<0.001)	0.65 (<0.001)
		➔	Dyskinesia	0.42 (0.012)	-	-	-	0.33 (0.007)	-
		➔	Fluctuations	0.33 (0.043)	-	-	-0.54 (<0.001)	-	-
		➔	Falls	0.78 (<0.001)	0.68 (<0.001)	0.60 (<0.001)	0.66 (<0.001)	0.77 (<0.001)	0.66 (<0.001)
		➔	Freezing	0.54 (-)	0.69 (-)	0.62 (-)	0.76 (-)	0.66 (-)	0.61 (-)
		➔	Hoehn & Yahr Stage	0.75 (<0.001)	0.58 (<0.001)	0.53 (<0.001)	0.46 (0.005)	0.72 (<0.001)	0.55 (<0.001)
	Non-Motor Domain	➔	Depression	0.91 (0.004)	0.78 (<0.001)	0.76 (0.004)	0.73 (0.018)	0.87 (<0.001)	0.78 (<0.001)
		➔	Anxiety	0.59 (0.011)	0.62 (<0.001)	0.63 (0.006)	0.49 (0.044)	0.65 (<0.001)	0.59 (<0.001)
		➔	Cognition	0.28 (0.014)	0.35 (<0.001)	0.23 (0.036)	0.33 (0.036)	0.25 (0.019)	0.42 (<0.001)
		➔	Hallucination	0.41 (-)	0.50 (-)	0.31 (-)	0.42 (-)	0.45 (-)	0.48 (-)
		➔	Apathy	0.55 (0.015)	0.43 (<0.001)	0.50 (0.010)	0.50 (0.042)	0.47 (0.002)	0.45 (0.003)
		➔	Fatigue	0.51 (0.018)	0.49 (<0.001)	0.49 (0.010)	-	0.49 (0.002)	0.52 (0.001)
		➔	Psychosocial Functioning	0.75 (0.006)	0.90 (<0.001)	0.83 (0.004)	0.75 (0.017)	0.86 (<0.001)	0.83 (<0.001)
		➔	Sleep Disturbances	-	0.33 (0.004)	0.32 (0.031)	-	-	0.37 (0.008)
		➔	Nutritional Status	-0.63 (0.010)	-0.62 (<0.001)	-0.56 (0.008)	-0.58 (0.030)	-0.69 (<0.001)	-0.58 (<0.001)
	HRQoL	➔	Mobility	0.90 (-)	0.84 (-)	0.71 (-)	0.83 (-)	0.89 (-)	0.80 (-)
		➔	ADL	0.83 (<0.001)	0.75 (<0.001)	0.55 (<0.001)	0.76 (<0.001)	0.78 (<0.001)	0.80 (<0.001)
		➔	Emotional Well-being	0.48 (<0.001)	0.65 (<0.001)	0.67 (<0.001)	0.50 (0.001)	0.70 (<0.001)	0.55 (<0.001)
		➔	Stigma	-	0.39 (<0.001)	0.33 (0.003)	-	-	0.49 (<0.001)
		➔	Social Behaviors	0.38 (0.007)	0.59 (<0.001)	0.46 (<0.001)	0.60 (<0.001)	0.61 (<0.001)	0.51 (<0.001)
		➔	Cognition	0.43 (0.002)	0.72 (<0.001)	0.64 (<0.001)	0.59 (<0.001)	0.61 (<0.001)	0.67 (<0.001)
		➔	Communication	0.69 (<0.001)	0.68 (<0.001)	0.54 (<0.001)	0.67 (<0.001)	0.79 (<0.001)	0.60 (<0.001)
		➔	Bodily Discomfort	0.43 (0.002)	0.61 (<0.001)	0.60 (<0.001)	0.61 (<0.001)	0.65 (<0.001)	0.48 (<0.001)

## Discussion

Capitalizing upon comprehensively evaluated variables and in addition to the broad list of motor and non-motor features, comorbidity profile and nutritional status were also included in our analysis, which have been mainly ignored in many previous studies as determinant factors of HRQoL in PD. HRQoL was found to be poorer in females, those with lower level of education, higher number of comorbid conditions and longer duration of disease. Interestingly, our findings showed that poorer nutritional status and less psychosocial activity also accompanied with poorer HRQoL in PD patients. The most critical determinants of HRQoL in Iranian PD patients were motor symptoms affecting their activities of daily life, depression, anxiety, and female sex, respectively. PD patients with a higher number of comorbidities had in average a 2.6 poorer score in cognitive dimension. Less severe motor signs, which is usually expected in the first stages of PD, associated with worse emotional-well being and stigma scores showing that these domains are mainly affected at the beginning of the disease and coping mechanisms during the next years could have improved these aspects of HRQoL.

*Wu et al* also reported that non-motor symptoms, higher motor severity shown by Hoehn and Yahr score and UPDRS-part III, motor complications, female sex, longer disease duration and being single or divorced all negatively affect the overall QoL in Chinese PD patients[6]. Their study population[6] was quite similar to ours regarding HRQoL demonstrated by the average PDSI (21.2 vs. 21.7), which have made these two studies comparable. In line with our findings, they have also shown that non-motor symptoms are the main drivers of worse HRQoL in all dimensions. They have used the non-motor symptoms scale of Parkinson's disease (NMSS) as a general indicator[6], while we have evaluated them with more details and shown that depression and anxiety are probably the main non-motor determinants of HRQoL in PD. So far, many other studies have also concluded that non-motor features were the main determinant factors for HRQoL in parkinsonian patients[1,2,3,4,6,20,21], and some have specified depression and anxiety as the main non-motor responsible for poor HRQoL[21,22,23,24,25]. ADL and the level of dependency have been commonly shown to be an important driver of worse HRQoL in PD[7,26], which is consistent with the independent role of UPDRS-ADL score in our investigation. Female sex as a risk factor for worse HRQoL in PD has been shown in some other studies[6], however, still debated with controversial findings in some other reports[2,5,27]. Although it is believed that the worse QoL in women with a chronic disease is generally resulted from the higher burden of depression and anxiety, our findings demonstrated that female sex is an independent risk factor for worsening of HRQoL in PD even after adjustment for psychiatric symptoms. In another comprehensive assessment on 130 PD patients, *Rahman et al* also showed that other than depression and anxiety, other non-motor symptoms such as fatigue, confusion, autonomic disturbance and pain and some motor problems specifically shuffling, difficulty in turning and dressing, falls and unpredictable fluctuations were the major predictors of worse HRQoL[25].

In addition to regression models, we also applied SEM consisted of global motor, non-motor and comorbidity components. Statistically, SEMs are stronger models due to the ability of complex linkage between different components through simultaneous regression equations, and taking into account interrelationships between predictor variables and observational errors from measurement of latent variables[28]. Our findings demonstrated that anxiety could directly strongly affect the emotional well-being domain of HRQoL and seemed to be its main determinant. Other than the already known indicators, our investigation revealed that nutritional status and fatigue played a key independent role in the non-motor component to affect HRQoL in PD. Previously, we have shown that PD patients with nutritional insufficiency had considerably worse HRQoL in all domains except for stigma [9]. Even in a longitudinal study by *Sheard et al*, improvement in nutritional status has demonstrated to advance HRQoL in patients with PD [8]. Most recently, weight loss has been also shown to worsen HRQoL, which is a common problem and should be noticed by practitioners [29]. These findings highlight the importance of the association between nutritional status and HRQoL in PD.

Motor and gait complications such as dyskinesia, falls, fluctuations and freezing were all significant indicators of the global motor component in its path to affect HRQoL. A considerable proportion of the overall contribution of motor symptoms in HRQoL mediated through non-motor component. In addition to this comprehensive general model, we also showed outstanding heterogeneities in the pattern of HRQoL in different PD phenotypes. Interestingly, the comorbidity component was an important determinant of HRQoL only among those with the older-onset and non-tremor-dominant PD. Among patients with rapid progression PD, a chronicity symptom such as fatigue was not a significant indicator of global non-motor section, while the motor component had a larger direct effect on HRQoL. In contrast, the impact of motor component on HRQoL was mostly mediated through non-motor symptoms as the main driver of HRQoL in slow-progression PD. Contribution of global motor component in HRQoL was remarkably different between the younger-onset and older-onset PD patients such that the importance of direct effects of motor symptoms on HRQoL was three-fold larger in younger-onset patients. Among those with the non-tremor dominant phenotype, the global non-motor component revealed larger contribution in HRQoL, which is in line with the findings from a recent study showing that non-motor symptoms had less involvement in HRQoL in PD patients with tremor-associated phenotype compared to those who predominantly manifested with axial symptoms [30].

So far, a few structural modelings have been performed to comprehensively elucidate HRQoL in PD. *Visser et al* have also constructed an SEM and concluded that psychosocial well-being had a larger impact on HRQoL than physical functioning among which depression had the largest contribution followed by axial motor, gastrointestinal, and urinary symptoms[31]. According to their model other impairments such as pain, psychiatric complications, motor symptoms, autonomic dysfunction, motor complications, and daytime sleepiness, indirectly affect HRQoL via psychosocial well-being and ADL[31]. *Soh et al* mainly highlighted the direct contribution of functional disability and falls in HRQoL of PD patients[32]. More recently, *Lee et al* have proposed another SEM in which depression and pain were introduced as the main factors that could directly affect HRQoL in PD[33]. Even though different structural models have been hypothesized, our findings on the general model are aligned with those of previous studies. In all SEMs of HRQoL, either functional disabilities or psychiatric well-being such as depression have been pointed out as the most consistent factors associated with poorer HRQoL in PD. Nevertheless, there are some differences in the list of variables that have been used to create these models and none of them have compared the model between different PD phenotypes.

We should acknowledge our study limitations of which the most important one is its cross-sectional design. Data on some other important features such as pain, and REM sleep behavior disorder were not available in our study. Data validity could have been improved by using more objective methods like polysomnography for sleep disturbances, blood pressure measurement to detect orthostatic hypotension and a full neuropsychological assessment for cognitive impairments. Still, the comprehensive list of variables and sophisticated statistical method we used, have strengthened our investigation. Similar to other previous studies on this topic, we should also mention that some of the items of the PDQ-39 questionnaire are inherently related to some non-motor symptoms such as depression, anxiety, sleep disorders and hallucinations, which might have led to the large contribution of non-motor component on HRQoL indicators.

In conclusion, our study provides a comprehensive understanding of HRQOL through the testing of conceptualized structural model, which has demonstrated global motor and non-motor components and their important indicators to affect HRQoL in addition to the comorbidity burden as the main drivers. Clear heterogenic HRQoL patterns were observed in patients with different phenotypes, which need to be taken into account for future studies. PD patients with younger-onset, older-onset, slow-progression, rapid-progression, motor-dominant, and non-motor-dominant phenotype have noticeably different causal pathways and determinants for HRQoL. These factors should be considered during the assessments and developing personalized strategies to improve HRQOL in PD patients with different phenotypes or prominent feature. Yet, it is necessary to examine the causal order of the determinants of HRQoL using data from longitudinal studies considering differences in the phenotypic features of PD.

## Supporting Information

S1 Text Datasheet containing detailed participant-level information on study variables. (XLSX)

## References

[1] Li H, Zhang M, Chen L, Zhang J, Pei Z, Hu A, et al (2010) Nonmotor symptoms are independently associated with impaired health-related quality of life in Chinese patients with Parkinson's disease. Mov Disord 25: 2740–2746. 10.1002/mds.23368 20945434

[2] Hinnell C, Hurt CS, Landau S, Brown RG, Samuel M (2012) Nonmotor versus motor symptoms: how much do they matter to health status in Parkinson's disease? Mov Disord 27: 236–241. 10.1002/mds.23961 21954027

[3] Martinez-Martin P, Rodriguez-Blazquez C, Kurtis MM, Chaudhuri KR (2011) The impact of non-motor symptoms on health-related quality of life of patients with Parkinson's disease. Mov Disord 26: 399–406. 10.1002/mds.23462 21264941

[4] Shearer J, Green C, Counsell CE, Zajicek JP (2012) The impact of motor and non motor symptoms on health state values in newly diagnosed idiopathic Parkinson's disease. J Neurol 259: 462–468. 10.1007/s00415-011-6202-y 21818689

[5] Winter Y, von Campenhausen S, Gasser J, Seppi K, Reese JP, Pfeiffer KP, et al (2010) Social and clinical determinants of quality of life in Parkinson's disease in Austria: a cohort study. J Neurol 257: 638–645. 10.1007/s00415-009-5389-7 19946784

[6] Wu Y, Guo XY, Wei QQ, Song W, Chen K, Cao B, et al (2014) Determinants of the quality of life in Parkinson's disease: results of a cohort study from Southwest China. J Neurol Sci 340: 144–149. 10.1016/j.jns.2014.03.014 24679837

[7] Soh SE, Morris ME, McGinley JL (2011) Determinants of health-related quality of life in Parkinson's disease: a systematic review. Parkinsonism Relat Disord 17: 1–9. 10.1016/j.parkreldis.2010.08.012 20833572

[8] Sheard JM, Ash S, Mellick GD, Silburn PA, Kerr GK (2014) Improved nutritional status is related to improved quality of life in Parkinson's disease. BMC Neurol 14: 212 10.1186/s12883-014-0212-1 25403709 PMC4237731

[9] Fereshtehnejad SM, Ghazi L, Shafieesabet M, Shahidi GA, Delbari A, Lökk J. (2014) Motor, psychiatric and fatigue features associated with nutritional status and its effects on quality of life in Parkinson's disease patients. PLoS One 9: e91153 10.1371/journal.pone.0091153 24608130 PMC3946796

[10] Langston JW (2006) The Parkinson's complex: parkinsonism is just the tip of the iceberg. Ann Neurol 59: 591–596. 16566021 10.1002/ana.20834

[11] Sieber BA, Landis S, Koroshetz W, Bateman R, Siderowf A, Galpern WR, et al (2014) Prioritized research recommendations from the National Institute of Neurological Disorders and Stroke Parkinson's Disease 2014 conference. Ann Neurol 76: 469–472. 10.1002/ana.24261 25164235 PMC5736367

[12] Hughes AJ, Daniel SE, Kilford L, Lees AJ (1992) Accuracy of clinical diagnosis of idiopathic Parkinson's disease: a clinico-pathological study of 100 cases. J Neurol Neurosurg Psychiatry 55: 181–184. 1564476 10.1136/jnnp.55.3.181 PMC1014720

[13] Gasparoli E, Delibori D, Polesello G, Santelli L, Ermani M, Battistin L, et al (2002) Clinical predictors in Parkinson's disease. Neurol Sci 23 Suppl 2: S77–78. 12548352 10.1007/s100720200078

[14] Montazeri A, Vahdaninia M, Ebrahimi M, Jarvandi S (2003) The Hospital Anxiety and Depression Scale (HADS): translation and validation study of the Iranian version. Health Qual Life Outcomes 1: 14 12816545 10.1186/1477-7525-1-14 PMC161819

[15] Fereshtehnejad SM, Hadizadeh H, Farhadi F, Shahidi GA, Delbari A, Lökk J. (2013) Reliability and validity of the persian version of the fatigue severity scale in idiopathic Parkinson's disease patients. Parkinsons Dis 2013: 935429 10.1155/2013/935429 24089644 PMC3780699

[16] Ghazi L, Fereshtehnejad SM, Abbasi Fard S, Sadeghi M, Shahidi GA, Lökk J. (2015) Mini Nutritional Assessment (MNA) is rather a reliable and valid instrument to assess nutritional status in Iranian healthy adults and elderly with a chronic disease. Ecol Food Nutr 54: 342–357. 10.1080/03670244.2014.994743 25714475

[17] Fereshtehnejad SM, Farhadi F, Hadizadeh H, Shahidi GA, Delbari A, Lökk J. (2014) Cross-cultural validity, reliability, and psychometric properties of the persian version of the scales for outcomes in Parkinson's disease-psychosocial questionnaire. Neurol Res Int 2014: 260684 10.1155/2014/260684 24804096 PMC3997069

[18] Nojomi M, Mostafavian Z, Shahidi GA, Jenkinson C (2010) Quality of life in patients with Parkinson's disease: Translation and psychometric evaluation of the Iranian version of PDQ-39. J Res Med Sci 15: 63–69. 21526061 PMC3082793

[19] Hooper D, Coughlan J, Mullen M (2008) Structural equation modelling: Guidelines for determining model fit. Electronic Journal of Business Research Methods 6: 53–60.

[20] Qin Z, Zhang L, Sun F, Fang X, Meng C, Tanner C, et al (2009) Health related quality of life in early Parkinson's disease: impact of motor and non-motor symptoms, results from Chinese levodopa exposed cohort. Parkinsonism Relat Disord 15: 767–771. 10.1016/j.parkreldis.2009.05.011 19553154

[21] Rodriguez-Violante M, Cervantes-Arriaga A, Corona T, Martinez-Ramirez D, Morales-Briceno H, Martínez-Martín P. (2013) Clinical determinants of health-related quality of life in Mexican patients with Parkinson's disease. Arch Med Res 44: 110–114. 10.1016/j.arcmed.2013.01.005 23376054

[22] Jones JD, Butterfield LC, Song W, Lafo J, Mangal P, Okun MS, et al (2014) Anxiety and Depression Are Better Correlates of Parkinson's Disease Quality of Life Than Apathy. J Neuropsychiatry Clin Neurosci 27: 213–218. 10.1176/appi.neuropsych.13120380 25162776 PMC4344415

[23] Kasten M, Kertelge L, Tadic V, Bruggemann N, Schmidt A, van der Vegt J, et al (2012) Depression and quality of life in monogenic compared to idiopathic, early-onset Parkinson's disease. Mov Disord 27: 754–759. 10.1002/mds.24999 22550041

[24] Quelhas R, Costa M (2009) Anxiety, depression, and quality of life in Parkinson's disease. J Neuropsychiatry Clin Neurosci 21: 413–419. 10.1176/appi.neuropsych.21.4.413 19996250

[25] Rahman S, Griffin HJ, Quinn NP, Jahanshahi M (2008) Quality of life in Parkinson's disease: the relative importance of the symptoms. Mov Disord 23: 1428–1434. 10.1002/mds.21667 18543333

[26] Hong SK, Park KW, Cha JK, Kim SH, Chun DY, Chang KW, et al (2002) Quality of life in patients with Parkinson’s disease. J Korean Neurol Assoc 2: 227–233.

[27] Zhao YJ, Tan LC, Lau PN, Au WL, Li SC, Luo N. (2008) Factors affecting health-related quality of life amongst Asian patients with Parkinson's disease. Eur J Neurol 15: 737–742. 10.1111/j.1468-1331.2008.02178.x 18494793

[28] Hancock G (2003) Fortune cookies, measurement error, and experimental design. J Mod Appl Stat Methods 2: 293–305.

[29] Akbar U, He Y, Dai Y, Hack N, Malaty I, McFarland NR, et al (2015) Weight loss and impact on quality of life in Parkinson's disease. PLoS One 10: e0124541 10.1371/journal.pone.0124541 25938478 PMC4418600

[30] Berganzo K, Tijero B, Gonzalez-Eizaguirre A, Somme J, Lezcano E, Gabilondo I, et al (2014) Motor and non-motor symptoms of Parkinson's disease and their impact on quality of life and on different clinical subgroups. Neurologia pii: S0213–4853(14)00233–3. 10.1016/j.nrl.2014.10.010 25529173

[31] Visser M, van Rooden SM, Verbaan D, Marinus J, Stiggelbout AM, van Hilten JJ. (2008) A comprehensive model of health-related quality of life in Parkinson's disease. J Neurol 255: 1580–1587. 10.1007/s00415-008-0994-4 18821041

[32] Soh SE, McGinley JL, Watts JJ, Iansek R, Murphy AT, Menz HB, et al (2013) Determinants of health-related quality of life in people with Parkinson's disease: a path analysis. Qual Life Res 22: 1543–1553. 10.1007/s11136-012-0289-1 23070750

[33] Lee J, Choi M, Jung D, Sohn YH, Hong J (2014) A Structural Model of Health-Related Quality of Life in Parkinson's Disease Patients. West J Nurs Res 37: 1062–1080. 10.1177/0193945914528588 24718037

